# Redox Characterization of Functioning Skeletal Muscle

**DOI:** 10.3389/fphys.2015.00338

**Published:** 2015-11-18

**Authors:** Li Zuo, Benjamin K. Pannell

**Affiliations:** ^1^Radiologic Sciences and Respiratory Therapy Division, School of Health and Rehabilitation Sciences, The Ohio State University College of MedicineColumbus, OH, USA; ^2^Interdisciplinary Biophysics Graduate Program, The Ohio State UniversityColumbus, OH, USA

**Keywords:** atrophy, redox, signaling, disease, oxidative stress

## Abstract

Skeletal muscle physiology is influenced by the presence of chemically reactive molecules such as reactive oxygen species (ROS). These molecules regulate multiple redox-sensitive signaling pathways that play a critical role in cellular processes including gene expression and protein modification. While ROS have gained much attention for their harmful effects in muscle fatigue and dysfunction, research has also shown ROS to facilitate muscle adaptation after stressors such as physical exercise. This manuscript aims to provide a comprehensive review of the current understanding of redox signaling in skeletal muscle. ROS-induced oxidative stress and its role in the aging process are discussed. Mitochondria have been shown to generate large amounts of ROS during muscular contractions, and thus are susceptible to oxidative stress. ROS can modify proteins located in the mitochondrial membrane leading to cell death and osmotic swelling. ROS also contribute to the necrosis and inflammation of muscle fibers that is associated with muscular diseases including Duchenne muscular dystrophy. It is imperative that future research continues to investigate the exact role of ROS in normal skeletal muscle function as well as muscular dysfunction and disease.

## Introduction

Current research is revealing new perspectives regarding the biological significance of free radicals and other chemically reactive molecules in numerous physiological processes and pathological conditions. Reactive oxygen species (ROS), when generated in excess amounts, contribute to oxidative stress. ROS produced from contracting skeletal muscle are known to affect both muscle adaption and function (Zuo et al., 2011a, 2013). However, these species, at optimal levels, can be critical for biological function. For instance, ROS have functional roles in innate and adaptive immunity, as well as initiating secondary signal transduction processes (Daiber, 2010; Nathan and Cunningham-Bussel, 2013; Zuo et al., 2014c). ROS can also cause structural and functional damage through lipid oxidation, and modification of proteins and DNA (Lobo et al., 2010). The increase of ROS has been considered a significant factor in the aging process as ROS contribute heavily to cellular oxidative stress, associated with aging (Hensley and Floyd, 2002; Liochev, 2013). Other potential effects exerted by ROS may include DNA damage, decreased mitochondria function, dysfunctional polypeptides, reduced muscle contractile force, and loss of muscle mass (Brandes et al., 2005; Shi et al., 2013). Particularly, ROS play an important role in the functioning of skeletal muscle (Zuo et al., 2013, 2015a), since ROS may mediate adaptive responses by facilitating glucose uptake or inducing mitochondrial biogenesis (Merry et al., 2010; Powers et al., 2011). On the other hand, research has also shown ROS to promote muscle fatigue and contribute to muscle dysfunction involved in a variety of chronic diseases (Reid and Moylan, 2011; Zuo et al., 2012).

## ROS/RNS in skeletal muscle

**Figure 1:**
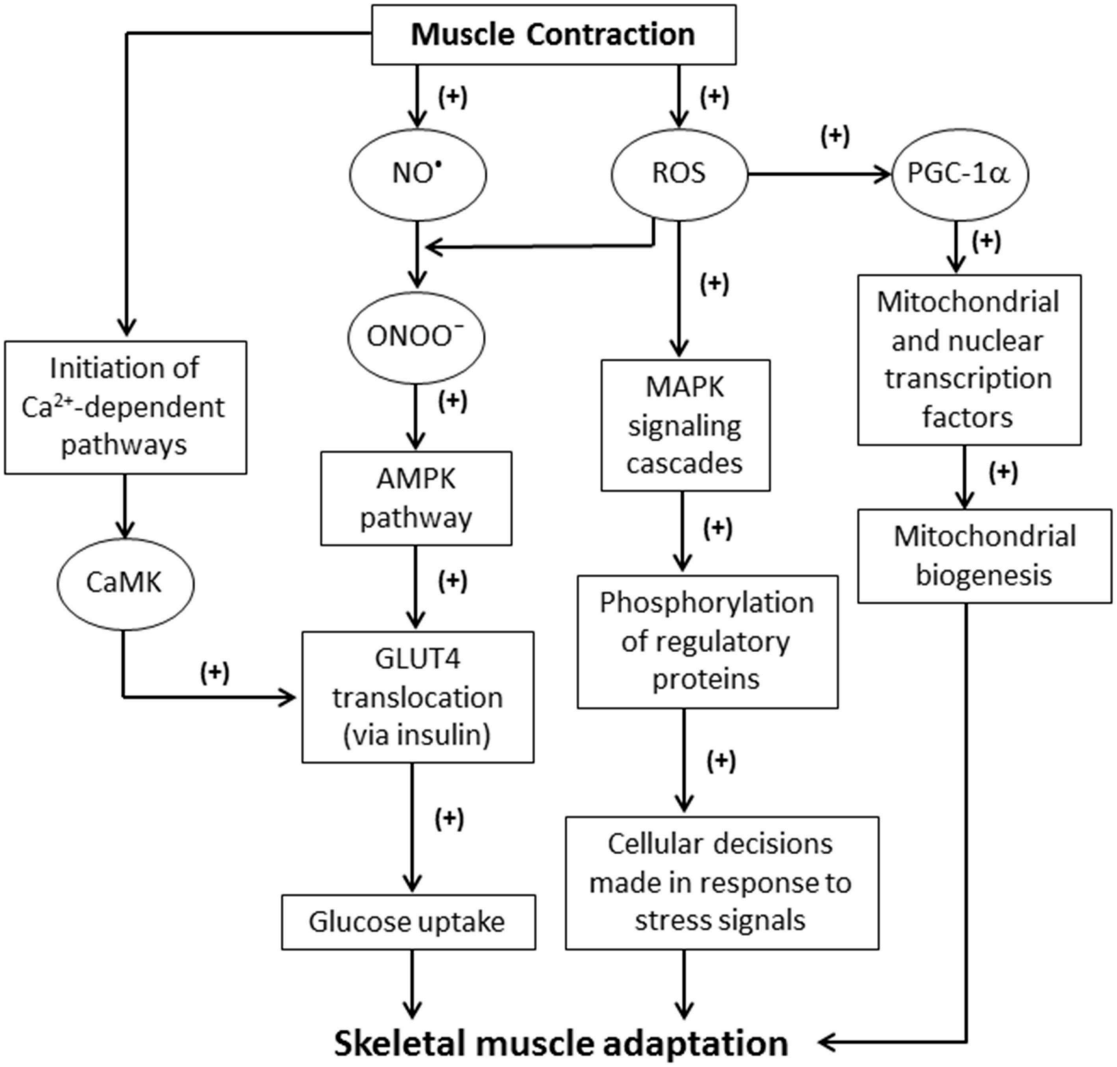
**Schematic illustrating the effects of ROS generated from skeletal muscle contractions**. NO, nitric oxide; ROS, reactive oxygen species; ONOO^−^, peroxynitrite; MAPK, mitogen-activated protein kinase; PGC-1α, proliferator-activated receptor-γ coactivator-1α; AMPK, 5′ adenosine monophosphate-activated protein kinase; GLUT4, glucose transporter type 4; CaMK, Ca^2+^/calmodulin-dependent kinase.

Sustained contractile activity of muscle fibers can result in inflammation, altered hormonal environments, and an increase in the production of ROS and other free radicals (Fittipaldi et al., 2014). Among these reactive molecules, nitric oxide (NO^•^) can be produced within skeletal muscle fibers from L-arginine by NO^•^ synthase (NOS). Three major isoforms of NOS exist: neuronal NOS (nNOS or NOS I); inducible NOS (iNOS or NOS II); and endothelial NOS (eNOS or NOS III) (Stamler and Meissner, 2001). NO^•^ production is vital for the mediation of signaling pathways during muscle contractions (Figure 1). For example, NO^•^ has been implicated in both the activation and inhibition of ryanodine receptors (RyR) (Hart and Dulhunty, 2000; Stamler and Meissner, 2001). RyR are redox sensitive channels that facilitate the rapid release of Ca^2+^ into the cytosol, which is critical for excitation-contraction coupling (Stamler and Meissner, 2001; Zalk et al., 2015). Hirschfield et al. reported that NO^•^ release increased eight-fold during active contractions in mouse diaphragmatic skeletal muscle (Hirschfield et al., 2000). Interestingly, this study also revealed neither NO^•^ release rates nor contractile function were affected in NOS III-deficient mice, suggesting that NOS III is not involved in muscle contractions (Hirschfield et al., 2000). This was unexpected given that NOS I and III are generally believed to be constitutively expressed in skeletal muscle (Hirschfield et al., 2000; Fleming, 2010). Further research may be required to explain the physiological relevance of NOS III in skeletal muscle. NOS I is found to be expressed in type II (fast-twitch) muscle fibers and is primarily located around the sarcolemma (Kobzik et al., 1994; Hart and Dulhunty, 2000). NOS II has been observed to be a marked source of NO^•^ production during immunological defense and inflammatory responses (Weinberg, 2000; Fleming, 2010). NO^•^ inhibition is associated with several disorders such as diabetes and hypertension (Cai and Harrison, 2000). One mechanism of NO inactivation is the reaction of NO^•^ with ${\text{O}}_{2}^{•-}$ that produces peroxynitrite (ONOO^−^), which is a potent oxidizing agent (Figure 1) (Hayashi et al., 2004; Sindler et al., 2009). The negative effects of such a reaction are compounded because of the increased levels of ROS and the corresponding NO^•^ deficiency.

Muscle contractions stimulate ${\text{O}}_{2}^{•-}$ formation through activation of NADPH oxidase (NOX) (Jackson, 2008), xanthine oxidase (XO) (Duncan et al., 2005; Gomez-Cabrera et al., 2010; Barbieri and Sestili, 2012), and several mitochondrial respiratory complexes and enzymes (Turrens, 2003). ${\text{O}}_{2}^{•-}$ can be further reduced to hydrogen peroxide (H_2_O_2_) via superoxide dismutases (SOD). However, H_2_O_2_ can, in turn, form highly reactive hydroxyl radicals (^•^OH) through a Fenton reaction (Thomas et al., 2009; Kothari et al., 2010). The newly formed ^•^OH chemically reacts with essential biological macromolecules such as carbohydrates, protein, and lipids leading to adverse functional alterations that are often irreversible (Wu and Cederbaum, 2003; Brieger et al., 2012).

## AMPK and ROS signaling

In skeletal muscle, ROS/RNS can play an important role in the regulation of glucose metabolism by activating the 5′ adenosine monophosphate-activated protein kinase (AMPK) pathway. ONOO^−^ can activate the muscular AMPK pathway and increase glucose metabolism (Xie et al., 2006). Additionally, stressors, such as hypoxia, may stimulate AMPK activation in order to produce ATP (Xie et al., 2006). AMPK is involved in the insulin-dependent translocation of glucose transporter type 4 (GLUT4), and consequently increases glucose uptake by myocytes (Figure 1). AMPK signaling also leads to ATP generation by the inhibition of acetyl-coenzyme A (CoA) carboxylase (ACC) (Chen et al., 2000; Schroeder et al., 2009; Wakil and Abu-Elheiga, 2009).

Increases in ROS production are generated through a variety of signaling mechanisms. For example, interleukin (IL)-13 plays a critical role in the immune system maintaining normal homeostasis as well as responding to pathogens (Mandal et al., 2010). IL-13 stimulates ROS as secondary messengers via janus kinase (JAK) signal transducer and activator of transcription (STAT) pathways (Mandal et al., 2010). IL-13 employs the MEK/ERK pathway to facilitate ROS production (Mandal et al., 2010). ROS also activate proliferator-activated receptor-γ coactivator-1α (PGC-1α) (via AMPK), nuclear factor κB (NF-κB), ERK 1/2, and p38 MAPK pathways (Irrcher et al., 2009; Mandal et al., 2010; Morris et al., 2013).

Increased ROS levels induce oxidative modification to the Met281/282 pair located in the regulatory domain of Ca^2+^/calmodulin-dependent kinase II (CaMKII) (Erickson et al., 2008, 2011). This suggests that ROS may indirectly have a role in skeletal muscle's ability to adapt to environmental stressors. Furthermore, ROS are involved in initiating the classical MAPK signaling cascade. The MAPK family inhibits or activates numerous signaling pathways through phosphorylation of regulatory proteins. This is a well-established mechanism by which cellular ROS levels induce skeletal muscle adaptation (Figure 1) (Cuschieri and Maier, 2005; Powers et al., 2010).

Interestingly, when untrained athletes begin intensive training, their immunological state is compromised due in part to the mitigation of neutrophilic ROS (Koga et al., 2013). ROS are also involved in angiotensin II-mediated physiological responses including interruption of endothelium-dependent vasodilation (Griendling and Ushio-Fukai, 2000). ROS mediated cell apoptosis is associated with the serine/threonine protein kinase mammalian target of rapamycin (mTOR) activity. The mTOR complexes can maintain cell homeostasis against stressors such as nutrient loss or growth factor deprivation. Dysregulation of mTOR activity impedes the cell's defense mechanism against growth factor deprivation, leading to endoplasmic reticulum stress (Sengupta et al., 2010), and apoptosis through overproduction of ROS (Ozcan et al., 2008; Di Nardo et al., 2009).

## Muscle adaptation and atrophy

ROS are known to be key mediators in redox signaling pathways stimulating cellular proliferation, growth, and differentiation (Ji, 2008). ROS generated during skeletal muscle contractions have been demonstrated to be critical to cell functionality (Ji, 2008; Barbieri and Sestili, 2012). Furthermore, the activity of factors such as nuclear factor of activated T-cells (NFAT), Ca^2+^/calmodulin-dependent kinase II (CaMKII) (the predominant CaMK isoform present in human skeletal muscle), and calcineurin all play a marked role in adaptation to events including exercise training (Chin, 2005; Erickson et al., 2011; Barbieri and Sestili, 2012; Ojuka et al., 2012). For example, the activation of CaMK is involved in the increase of glucose transport activity via GLUT4 translocation during contractile activity (Figure 1) (Wright et al., 2004; Hawley et al., 2006; Egan and Zierath, 2013). The inhibition of CaMK significantly reduces glucose uptake *in vitro* (Hawley et al., 2006).

ROS trigger pathways that result in skeletal muscle remodeling and protein synthesis (Powers et al., 2010). Skeletal muscle remodeling serves as an adaptive response that enables the muscle to accommodate physiological changes (Bassel-Duby and Olson, 2006). ROS production is necessary for the remodeling of skeletal muscle in strenuous exercise (Barbieri and Sestili, 2012). Indeed, research has indicated that increased ROS production is a necessary response to exercise of a sufficient intensity (Vollaard et al., 2005). However, there is also evidence that ROS are involved in skeletal muscle damage and dysfunction after intense or unaccustomed exercise (Close et al., 2004). One study investigated the effects of manganese SOD (Mn-SOD) deficiency in mice and found that these mice were functionally impaired during intense, but not mild, exercise (Kuwahara et al., 2010). Mn-SOD serves as protection for the mitochondrial matrix by converting ${\text{O}}_{2}^{•-}$ into H_2_O_2_. However, skeletal muscle atrophy was not found to be a consequence of minimized Mn-SOD activity (Kuwahara et al., 2010; Lustgarten et al., 2011). Disrupted ROS homeostasis in the myoplasm is proposed to be a central cause of muscle fatigue (Kuwahara et al., 2010). Moderate exercise is not associated with the oxidative stress-induced damage in the same way as exhaustive exercise (Gomez-Cabrera et al., 2008). Instead, it is believed that moderate, regular exercise stimulates optimal ROS production that upregulates the expression of antioxidant defense systems (Gomez-Cabrera et al., 2008). Regular exercise reduces a person's risk for oxidative stress-related cardiovascular diseases, cancer, or stroke (Radak et al., 2005). Therefore, moderate levels of oxidant production, as a result of exercise, may confer biological protection (Radak et al., 2005; Gomez-Cabrera et al., 2008). Alternatively, during extended periods of disuse, redox imbalances contribute to deleterious skeletal muscle remodeling (e.g., atrophy) via myonuclear apoptosis and muscle atrophy (Powers et al., 2010). Interestingly, NF-κB, a redox sensitive transcription factor, may regulate as many as 150 genes during muscle adaptation in response to inactivity or exercise (Sun et al., 2002; Bassel-Duby and Olson, 2006; Powers et al., 2010). One study investigated the activity of pathways that respond to chronic muscle disuse induced by denervation. Increased ROS production played a large role in mediating muscle atrophy and weakness, lowering mitochondrial content, and increasing apoptosis signaling (Table 1) (Adhihetty et al., 2007). A different study reported a 30-fold increase in mitochondrial ROS production 7 days after denervation in skeletal muscle (Muller et al., 2007). This finding corroborated a strong correlation between muscle atrophy and ROS production. Apoptosis also contributes to muscle degeneration during normal aging and pathological conditions (Adhihetty et al., 2007). For instance, skeletal muscle atrophy is a consequence of many systemic diseases (e.g., cancer, diabetes, renal failure) that can cause cell death (Lecker et al., 2004). It is suspected that the underlying oxidative stress present in multiple chronic diseases leads muscle fibers into a catabolic state and subsequent wasting (Moylan and Reid, 2007).

**Table 1:** **Detrimental effects of ROS in skeletal muscle**.

**Process**	**Mechanism**	**Effect**	**References**
Aging myocytes	Oxidation of mtDNA	Reduced capability of mitochondria to produce cellular energy	Picard et al., 2014
	mPTP opening	Mitochondrial swelling Escape of proapoptotic proteins Apoptotic or necrotic cell death	Lemasters et al., 2009; Barbieri and Sestili, 2012
	AIF and Endo G release	DNA fragmentation	Barbieri and Sestili, 2012
	Induced activity of proteases and nucleases	Apoptosis	Cadenas and Davies, 2000
Skeletal muscle contraction	Activation of calpains and caspases	Myofibrillar protein degradation	Ochala et al., 2011
	Nitrosylation of RyR1 receptor	Increase the leakiness of sarcoplasmic reticulum Ca^2+^	Bellinger et al., 2009; Westerblad and Allen, 2011
	Oxidation of sarcolemmal lipids or contractile proteins	Muscle dysfunction	Goldstein and Mcnally, 2010
Neuromuscular function	SOD1 deficiency	Motor axon degeneration	Fischer et al., 2011, 2012
Chronic muscle disuse	Decreased mitochondrial content; increased ROS production and apoptosis signaling	Muscle atrophy and weakness	Adhihetty et al., 2007

*AIF, apoptosis inducing factor; Endo G, mitochondrial endonuclease G; mPTP, mitochondrial permeability transition pore; mtDNA, mitochondrial DNA; SOD1, Cu, Zn-superoxide dismutase*.

## ROS, mitochondria, and aging

Mitochondria are viewed as one of the major contributors of ROS production (Zuo et al., 2011b). *In vivo* experimentation has confirmed that increased oxidative stress impairs mitochondrial function (Williams et al., 1998; Berneburg et al., 1999). ROS can also target proteins in the mitochondrial membrane and lead to mitochondrial permeability transition (MPT) (Cosso et al., 2002). ROS can contribute to the opening of the mitochondrial permeability transition pore (mPTP) (Lemasters et al., 1998). One such mechanism of ROS-mediated mPTP opening is by the oxidation of dithiols in the protein pore located on the inner mitochondrial membrane (Lemasters et al., 1998). The mPTP is very sensitive to pathological conditions such as mitochondrial autophagy, tissue ischemia, and infarction (Lemasters et al., 1998, 2009). Prolonged mPTP opening causes the disruption of osmotic balance that drives mitochondrial swelling and ultimately leads to apoptotic or necrotic cell death (Table 1) (Lemasters et al., 2009). This is physiologically significant in photodynamic therapy in which targeted mitochondrial damage leads to cancer cell apoptosis (Kowaltowski et al., 2009).

Interestingly, certain ROS (e.g., H_2_O_2_) affect mtDNA more extensively than nuclear DNA (Berneburg et al., 1999). Mitochondrial complexes I and III are the primary source of ${\text{O}}_{2}^{•-}$ production (Babu et al., 2015). Due to the proximity to mtDNA, higher levels of ROS production are associated with more frequent mtDNA rearrangements (Esposito et al., 1999; Kirkinezos and Moraes, 2001). Moreover, ·OH, the product of H_2_O_2_ in Fenton reactions, may damage multiple molecular structures including mtDNA (Kirkinezos and Moraes, 2001). The capability of mitochondria to produce cellular energy is diminished with MPT, long-term fragmentation, and accumulation of damaged mtDNA (Table 1) (Picard et al., 2014). Oxidized mtDNA may also be released along with other immunogenic molecules, further inducing inflammation and modify gene expression (Picard et al., 2014).

Exercise-induced mitochondrial biogenesis may lead to a wide range of health benefits, including improved exercise tolerance, oxidative capacity, and insulin resistance (Powers et al., 2011). Mitochondrial biogenesis requires a synergistic effort between both mitochondrial and nuclear genomes, as well as coordinated responses from a variety of transcription factors and co-activators (Hood, 2001; Adhihetty et al., 2003; Powers et al., 2011). Both peroxisome proliferator-activated receptor-γ (PPAR-γ) and PGC-1α are actively involved in the regulation of mitochondrial biogenesis (Irrcher et al., 2009; Powers et al., 2011). PGC-1α also interacts with nuclear receptors and transcription factors that upregulate genes, including estrogen-related receptor-α (ERR-α) and myocyte enhance factor (MEF), contributing to organelle synthesis (Powers et al., 2011).

The expression of PGC-1α is influenced by a variety of stimuli that coincide with muscular exercise (Powers et al., 2011). Intracellular redox status appears to significantly impact PGC-1α activity (Powers et al., 2011). For instance, increased levels of exogenous ROS have been shown to stimulate PGC-1α (Irrcher et al., 2009), while the antioxidant N-acetyl cysteine may hinder such activation (Powers et al., 2011). The PGC-1α promoter contains transcription factor-binding sites such as activating transcription factor 2, tumor suppressor gene p53, enhancer box (EBox) proteins, MEF-2, and erythroid transcription factor (GATA), many of which are ROS-sensitive (Powers et al., 2011). Thus, exercised-induced elevation in ROS levels and the subsequent PGC-1α activation may lead to the upregulation of mitochondrial and nuclear DNA transcription factors (Figure 1).

ROS initiate degradative processes leading to oxidative stress presented in the forms of modified proteins and nucleic acids (Cadenas and Davies, 2000). These damaged cellular components are then degraded by proteases or nucleases, ultimately leading to oxidative stress-induced apoptosis (Cadenas and Davies, 2000). These harmful events play a significant role in the aging process (Table 1) (Cadenas and Davies, 2000). For instance, disruptions in redox homeostasis are associated with aging in various tissues, including skeletal muscle (Carter et al., 2007; Meng and Yu, 2010). Rats possess a reduced oxidative capacity in aging skeletal muscle (Hepple et al., 2003). ROS are associated with sarcopenia, defined as the loss of muscle mass and function (Shahar et al., 2013). The incidence of sarcopenia is growing and affects 11–50% of individuals over the age of 80 (von Haehling et al., 2010). Systemic inflammation, diminished ability to respond to stressors, and weakened regenerative capacity are all associated with sarcopenia (Lightfoot et al., 2014). Restoring oxidative balance may prevent or delay the onset of sarcopenia (Lightfoot et al., 2014). For example, resveratrol reduces oxidative stress in skeletal muscle (Jackson et al., 2010; Ryan et al., 2010). However, resveratrol is ineffective in attenuating sarcopenia (Jackson et al., 2011). Therefore, the mechanism underlying the link between oxidative stress and sarcopenia remains to be elucidated. Nonetheless, the aging process leads to a decline of such redox-mediated adaptations essential for skeletal muscle conditioning—contributing to age-related deterioration (Jackson, 2011).

Impaired mitochondrial function and relative ROS increases may occur in aging myocytes (Crescenzo et al., 2015). Although acute increases in ROS can improve muscle contractile endurance, long-term exposure to elevated amounts of ROS provoke proteolysis and cell death in skeletal muscle (Powers et al., 2010). Recent evidence indicates that ROS are involved in apoptosis by oxidizing crucial cellular components (e.g., proteins, nucleic acids, and lipids) (Barbieri and Sestili, 2012). ROS serve as cellular intermediates in apoptosis by interfering with the sarcoplasmic reticulum Ca^2+^ ion flux, resulting in the activation of calpain and caspase-7 (Barbieri and Sestili, 2012). Moreover, ROS can induce mitochondrial swelling and fragmentation, facilitating the opening of mPTPs and the escape of proapoptotic proteins (e.g., cytochrome *c*) (Table 1) (Barbieri and Sestili, 2012). ROS induce the release of apoptosis inducing factor (AIF) and mitochondrial endonuclease G (Endo G), resulting in DNA fragmentation in skeletal muscle (Table 1) (Barbieri and Sestili, 2012). Accordingly, Muller et al. suggested that the mechanism underlying skeletal muscle atrophy is tightly associated with ROS overproduction in the aging mitochondria (Muller et al., 2007).

## Redox in neuromuscular degeneration

Since neurons continuously generate free radicals, a sufficient supply of endogenous antioxidants is necessary to maintain homeostasis (Galea et al., 2012). Nerve cells are vulnerable to the damaging effects of oxidative stress including the oxidation of cytoskeletal proteins (Carletti et al., 2011). Oxidative stress plays a significant role in neuromuscular diseases such as amyotrophic lateral sclerosis (Barber et al., 2006; Fischer et al., 2012). Deficiency in one of the major antioxidants, Cu, Zn-superoxide dismutase (SOD1), may induce motor axon degeneration (Table 1) (Fischer et al., 2011, 2012). Additionally, the accumulation of very long chain fatty acids (VLCFA) in neurodegenerative disorders may result in electron chain impairment, thereby leading to ROS formation and lipid peroxidation. One of the main neurodegenerative disorders caused by VLCFA is the X-linked adrenoleukodystrophy (X-ALD) (Kruska et al., 2015), associated with the loss of peroxisomal ABCD1 fatty-acid transporter function and VLCFA accumulation (Galea et al., 2012).

## Skeletal muscle dysfunction

ROS are involved in the modulation of skeletal muscle contractions (Zuo et al., 2014a, 2015b). However, excessive oxidants promote muscle fatigue (Reid and Moylan, 2011). Evidence has shown that muscle-derived oxidants are significantly involved in tumor necrosis factor-α (TNF-α)-related loss of force (Reid and Moylan, 2011; Zuo et al., 2014b). Myofibrillar protein degradation, mediated by calpains and caspases, may be activated by upstream factors including ROS (Table 1) (Ochala et al., 2011). The overproduction of ROS, via NOX activation, is also seen in diabetic skeletal muscle. In particular, ROS-mediated glucose uptake may further contribute to the elevated oxidative stress (Ebrahimian et al., 2006; Barbieri and Sestili, 2012; Sinha et al., 2013). Additionally, ROS have been implicated in the pathogenesis of muscular dystrophies such as Duchenne muscular dystrophy (DMD) (Tidball and Wehling-Henricks, 2007). Dystrophin is one of the major proteins designed for the integrity of skeletal muscle fibers. Dysfunction or absence of dystrophin molecules can cause muscular diseases such as DMD, associated with necrosis of skeletal muscle fibers, as well as progressive muscular atrophy (Tidball and Wehling-Henricks, 2007). There have been multiple studies that disclose a link between ROS and DMD. For example, ROS can stimulate the production of proinflammatory cytokines including TNF-α and IL-1β through the activation of the NF-κB pathways (Malik et al., 2012). These inflammatory cytokines are significantly increased in dystrophic muscle cells immediately preceding muscle fiber necrosis (Malik et al., 2012). ROS also interfere with the functioning of RyR1. As such, nitrosylation of RyR1 receptors via NOS can increase the leakiness of sarcoplasmic reticulum Ca^2+^ associated with muscular dystrophy (Table 1) (Bellinger et al., 2009; Westerblad and Allen, 2011). ROS oxidize sarcolemmal lipids or contractile proteins, contributing to muscle dysfunction (Table 1) (Goldstein and Mcnally, 2010).

## Conclusion

The research reviewed in this article has identified many of the reactive species that affect skeletal muscle function. The pathways mediated by ROS/RNS signaling have been discussed as well as their role in skeletal muscle physiology. ROS have both beneficial and harmful effects on skeletal muscle function as they are ubiquitously involved in gene expression, immune responses, mitochondrial oxidative stress, skeletal muscle atrophy, as well as neuromuscular dystrophies. Redox-sensitive pathways continue to be an area of much needed research. Such studies in the future will shed significant insight into the exact role of ROS in health and disease.

## Author contributions

LZ designed the outline of the paper and wrote the paper; BP wrote the paper.

## Funding

This work was supported by OSUCOM-HRS Fund 013000.

### Conflict of interest statement

The authors declare that the research was conducted in the absence of any commercial or financial relationships that could be construed as a potential conflict of interest.
